# Distinguishing between plasmon-induced and photoexcited carriers in a device geometry

**DOI:** 10.1038/ncomms8797

**Published:** 2015-07-13

**Authors:** Bob Y. Zheng, Hangqi Zhao, Alejandro Manjavacas, Michael McClain, Peter Nordlander, Naomi J. Halas

**Affiliations:** 1 Department of Electrical and Computer Engineering, Rice University, 6100 Main St., Houston, TX 77005, USA; 2 Laboratory for Nanophotonics (LANP), Rice University, 6100 Main St., Houston, TX 77005, USA; 3 Department of Physics and Astronomy, Rice University, 6100 Main St., Houston, TX 77005, USA; 4 Department of Chemistry, Rice University, 6100 Main St., Houston, TX 77005, USA

## Abstract

The use of surface plasmons, charge density oscillations of conduction electrons of metallic nanostructures, to boost the efficiency of light-harvesting devices through increased light-matter interactions could drastically alter how sunlight is converted into electricity or fuels. These excitations can decay directly into energetic electron–hole pairs, useful for photocurrent generation or photocatalysis. However, the mechanisms behind plasmonic carrier generation remain poorly understood. Here we use nanowire-based hot-carrier devices on a wide-bandgap semiconductor to show that plasmonic carrier generation is proportional to internal field-intensity enhancement and occurs independently of bulk absorption. We also show that plasmon-induced hot electrons have higher energies than carriers generated by direct excitation and that reducing the barrier height allows for the collection of carriers from plasmons and direct photoexcitation. Our results provide a route to increasing the efficiency of plasmonic hot-carrier devices, which could lead to more efficient devices for converting sunlight into usable energy.

## Abstract (web summary)

Plasmonic excitations of electrons in metallic nanostructures are promising for the enhanced conversion of light in semiconductor solar cells. Here, the authors are able to experimentally distinguish the absorption phenomena of plasmonic carrier generation and excitation of carriers by light absorption.

## Graphical abstract

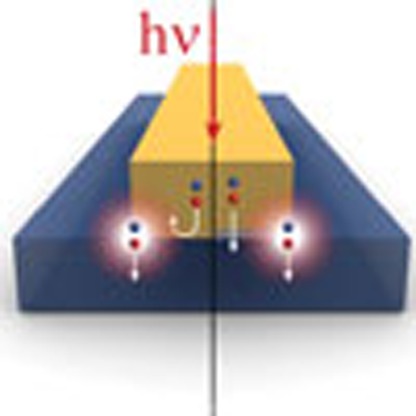

The use of metal nanoparticles and nanostructures for enhanced solar energy conversion has shown to be a promising route towards direct light-to-fuel synthesis, or more efficient photovoltaic devices^1^,^2^,^3^,^4^. Still, a full understanding of the mechanisms of plasmonic carrier generation remains elusive and many groups have sought to understand the vast body of experimental work^5^,^6^,^7^,^8^,^9^,^10^,^11^,^12^,^13^,^14^,^15^,^16^,^17^,^18^,^19^ by calculating hot-carrier efficiencies through *ab initio* calculations^20^,^21^,^22^,^23^. Two possible hot-carrier-generation mechanisms in metals are direct photoexcitation and plasmon decay. Hot-carrier generation by direct photon absorption is possible in metallic structures, but the small electron–photon cross-section makes this process fundamentally very inefficient. The efficiency can be somewhat compensated for in a metal with a larger density of electronic states, as in the case of the d-band electrons in Au, but it is ultimately limited by the inherent optical absorption in the metal. On the other hand, plasmonic nanostructures exhibit extremely large absorption cross sections, which can be significantly larger than the physical cross section of the nanostructure. Furthermore, plasmonic absorbers can obtain near-perfect absorption^24^, indicating that plasmon-induced hot-carrier generation could be extremely efficient.

Since plasmonic hot-carrier generation depends fundamentally on photon absorption, previous work has focused on correlating the experimentally measured photocatalytic activity spectrum or photocurrent responsivity with the calculated absorption spectrum^1^,^2^. Optical absorption is a local quantity that can be calculated by integrating the product of frequency *ω*, local electric field strength |**E**|^*2*^ and the imaginary part of the dielectric permittivity over the full volume of the nanostructure. However, hot carriers generated far from the nanoparticle surface can undergo scattering, recombination or lose energy in other ways and ultimately prevent its use in chemical reactions or injection over an energy barrier. Only carriers generated closer to the interface than the mean-free path, *l*_MFP_, can participate in interfacial electron transfer^12^. Thus, the relevant quantity for electron transfer is:


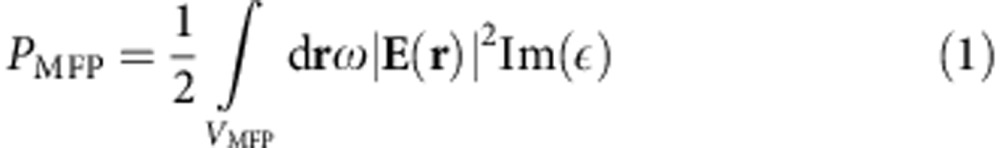


where *V*_MFP_ is the volume within a distance of *l*_MFP_ from the active interface. However, this type of analysis cannot distinguish between directly photoexcited carrier generation and high-energy carriers generated from plasmon decay, an important distinction in many applications. In photocatalysis, where chemical transformation is induced by the injection of hot carriers over an energy barrier into an unoccupied molecular orbital of an adsorbate molecule, it is of vital importance to understand which carrier-generation process can ultimately lead to useful, high-energy hot carriers.

For photocurrent generation, hot-carrier extraction generally involves injecting hot carriers over a Schottky barrier^25^,^26^. A Schottky barrier is formed at a metal–semiconductor junction and only allows significant current flow in one direction. Furthermore, Schottky diodes are majority-carrier devices where the current flow is conducted by either electrons or holes, but not both. This allows a Schottky diode to exclusively collect either hot electrons or hot holes, and prevents recombination, minimizing current loss. In this work, we exploit the material properties of reduced TiO_2_, which preferentially transports electrons^27^, to compare the properties of electrons collected across Ohmic junctions, where the effective barrier height is essentially zero, with those collected across a Schottky barrier.

## Results

### Schottky versus Ohmic interfaces

**Figure 1:**
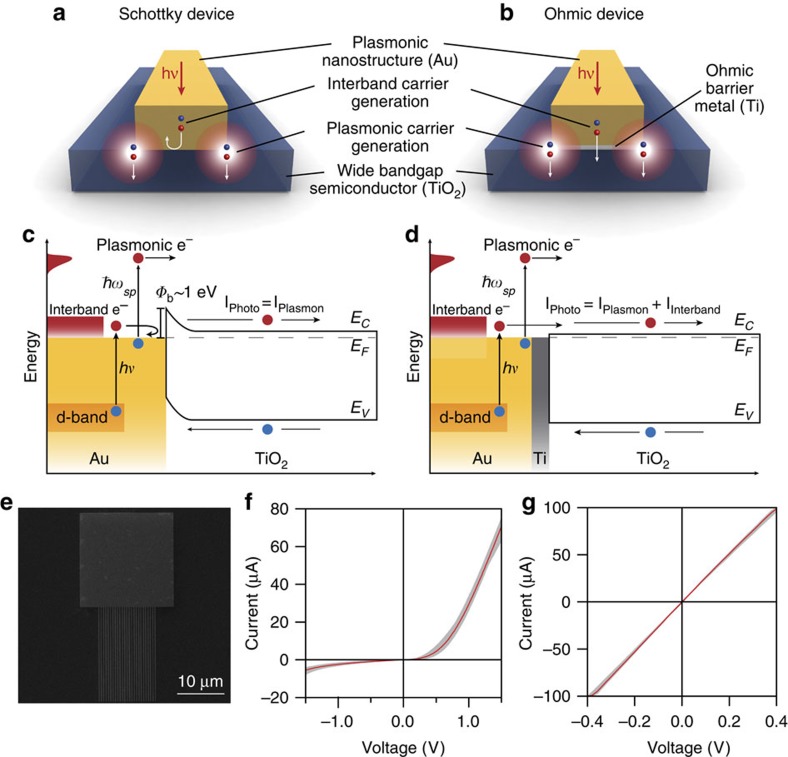
Device overview and band diagrams. Schematic of hot-carrier generation and collection over a Schottky (**a**) or an Ohmic barrier (**b**). Plasmonic hot-carrier generation from surface plasmons is localized to areas of large field enhancements, while hot carriers generated from interband absorption can occur throughout the bulk material, limited instead by absorption depth. Band diagram schematics of (**c**) a Au-TiO_2_ Schottky device and (**d**) a Au-Ti-TiO_2_ Ohmic device. Carrier generation by direct photoexcitation results from the excitation of d-band electrons, 2.3 eV below the Fermi level, into the conduction band. Their low energy prevents them from crossing the Schottky barrier (∼1 eV). Ohmic devices have no effective barrier and allows for collection of carriers created by this process. The wide bandgap of the semiconductor allows preferential collection of electrons. (**e**) Representative scanning-electron microscope (SEM) image of a fabricated nanostructure comprised of a contact pad and a nanowire array. Current-voltage (I–V) curves of Schottky (**f**) and Ohmic devices (**g**). Red curves are the averages and all measured I–V curves fall within the grey bounded regions.

The different properties of Schottky and Ohmic contacts allow us to selectively probe the two different carrier-generation mechanisms in metals (Fig. 1a,b). With a Schottky contact, we expect to collect only hot carriers generated from plasmon decay. This is because directly photoexcited carriers in Au are generated primarily from interband transitions and excited from the d-band with its upper edge ∼2.3 eV below the Fermi level^28^. For optical excitations from 1 to 3 eV, electrons are excited from the d-band to a maximum of ∼0.7 eV above the Fermi energy (Fig. 1c,d). The same type of electronic excitations can occur in plasmon decay^23^. However, the physical mechanism underlying electron–hole pair generation in plasmon decay is different than in direct excitation from incident plane waves. In plasmon-induced carrier generation, the perturbing potential driving the transitions is the plasmon-induced near field, which is localized to the surfaces of nanostructures. Plasmon-induced carrier generation thus favors excitation of electrons from near the Fermi energy, resulting in substantially higher-energy electrons^22^. For a Schottky barrier height of 1 eV, only the high-energy electrons will have sufficient kinetic energy to overcome the barrier. Therefore, the net photocurrent is exclusively from plasmon decay (Fig. 1b). For an Ohmic contact, where no barrier is present, low-energy electrons can also be collected, so the net photocurrent should have both plasmonic and interband contributions (Fig. 1d). While it is typically assumed that carrier separation in plasmonic hot electron devices requires an electric field^25^,^29^, the band alignment of the Ohmic device presents electrons and holes with two very different barrier heights. For electrons, the Ti-barrier layer forces the Fermi level of gold to align with the conduction band of TiO_2_. However, this band alignment results in a very large barrier height for holes and allows the TiO_2_ to efficiently and preferentially collect photogenerated electrons.

This study helps resolve a fundamental question in surface plasmon photophysics by demonstrating the large energy difference between hot carriers produced by surface plasmons and interband transitions. This large energy difference allows for a theoretical framework that largely ignores the band structure of the metal and focuses instead on field-intensity enhancement. Our study further demonstrates that it is possible to collect both plasmonic and interband photocurrents without a rectifying barrier and shows a surprising deviation from the commonly observed Fowler response in silicon-based devices^25^.

### Device geometry

We designed a simple device geometry consisting of a square metal pad and a metal nanowire array fabricated onto a TiO_2_ substrate (Fig. 1e). All nanostructures were 50-nm thick and adjacent nanowires were spaced 500-nm apart for all devices. Arrays of devices of varying nanowire widths were fabricated on single-crystal rutile <100> TiO_2_ substrates using standard cleanroom and electron-beam lithography techniques. The substrates initially exhibited extremely high resistance (>100 GΩ). Heating the substrates in vacuum introduced oxygen vacancies, n-doping the crystal substrate^30^. The resistance across the crystal decreased to ∼10 kΩ after heat treatment and the crystal colour changed from slight yellow to blue (Supplementary Fig. 1). This colour change is due to free carrier absorption from shallow mid-band trap states^30^, which does not increase the number of free carriers under illumination and therefore, does not contribute to photocurrent. These trap states do not alter the bandgap of TiO_2_ and serve as the main mechanism for conducting electrons across the substrate.

Ohmic and Schottky devices were patterned and fabricated on the same TiO_2_ substrate using electron-beam lithography and shadow masking. Au-TiO_2_ junctions form Schottky contacts while Au/Ti/TiO_2_ junctions form Ohmic contacts. The Ti-barrier layer is 2 nm and kept thin to minimize plasmon damping. The Methods section covers the fabrication process in more detail.

### Electrical characterization

The current-voltage (I-V) characteristics for the two devices are shown in Fig. 1f,g. The red line is the average device current-voltage (I-V) curve and all device I-V curves are bounded by the grey regions. The Schottky devices exhibit current rectification while Ohmic devices show linear I-V characteristics. We extracted Schottky barrier heights by fitting the I-V curves with the diode equation^31^ and obtained barrier heights between 1.02 and 1.13 eV, with an average of 1.07 eV. These measurements agree well with previous reports of Au-TiO_2_ Schottky barrier heights (∼1 eV)^32^. A lock-in amplifier was used for photocurrent measurements; all devices were measured without an applied bias voltage. We verified that the measured photocurrent in the Ohmic devices is consistent with electron injection, and not explained by changes in device conductance or junction resistances (Supplementary Fig. 2). In addition, we verified that photocurrent losses due to charge recombination in the substrate were minimal (Supplementary Fig. 3).

### Photocurrent mapping

**Figure 2:**
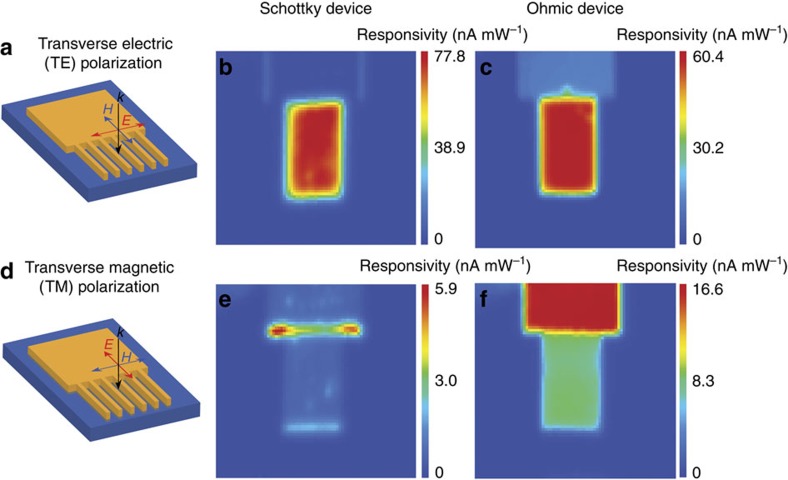
Photocurrent mapping. Schematic of TE (**a**) excitations used to generate photocurrent maps. The laser wavelength is tuned to the resonance of the plasmonic nanowires (∼675 nm, wire width 273 nm). Photocurrent maps of a (**b**) Schottky and (**c**) an Ohmic device using TE-polarized light. (**d**) Schematic of TM- polarized light excitation. Photocurrent maps of a (**e**) Schottky and (**f**) an Ohmic device. In the Schottky device, photocurrent production is drastically reduced while in the Ohmic device, photocurrent is observed throughout the metal nanostructure and in the nanowires.

Mapping the photocurrent as a function of polarization of the incident light in the various regions of each device allows us to determine the specific regions of the structure where the photocurrent originates, which allows us to discriminate between the plasmon-induced and directly photoexcited current contributions. We produce photocurrent maps by raster scanning a diffraction-limited laser spot (∼3 μm spot size) over a device and using a lock-in amplifier to record the photocurrent signal. The substrate produces no photocurrent since the wavelength of the incident light, 675 nm, which corresponds to the plasmon excitation energy of 1.84 eV, cannot be directly absorbed by rutile TiO_2_ (bandgap ∼3.03 eV)^33^. For light polarized transverse to the plasmonic nanowires (TE polarization), shown schematically in Fig. 2a, we observe photocurrent generation in the Schottky device (Fig. 2b) when scanning over the plasmonic nanowires and at points of broken symmetry along the edges of the metal pad. In the Ohmic device (Fig. 2c), photocurrent is produced when scanning over the nanowires as expected, but photocurrent is also produced throughout the entire pad region, where no plasmon mode exists (Supplementary Fig. 4). The spatial distribution of this photocurrent generation suggests that it arises from bulk absorption, since the photocurrent is generated homogeneously throughout the pad area. We also generated photocurrent maps using transverse-magnetic (TM) polarized light (Fig. 2d). For the Schottky device (Fig. 2e), no photocurrent is generated in the substrate and little photocurrent is produced by the metal nanostructure except at point defects and edges. In stark contrast, a significant amount of photocurrent is observed throughout the Ohmic device (Fig. 2f) in the pad region, as well as in the nanowires, for which no plasmon mode is excited. This photocurrent, which arises regardless of polarization and geometry, shows that the photocurrent in the Ohmic devices results from direct excitation and not plasmon decay, where the photocurrent would exhibit a strong dependence on the geometry of the metal nanostructure, as well as the polarization of incident light.

### Device responsivity

**Figure 3:**
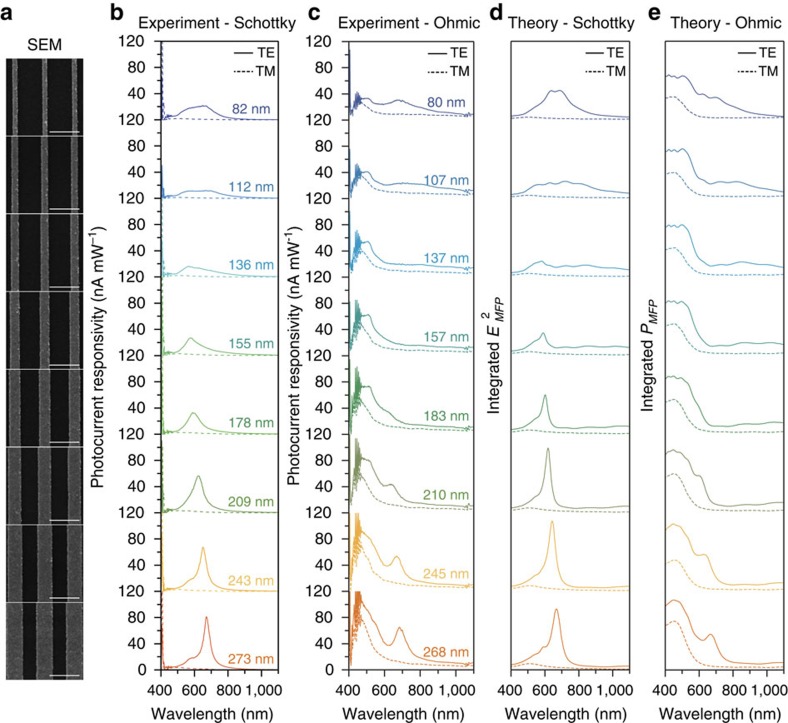
Device responsivities. (**a**) SEM images of the different nanowire widths used for responsivity measurements. Scale bar, 500 nm for all images. (**b**) Experimentally measured responsivities for Schottky devices when excited with TE (solid) and TM (dashed) polarizations. (**c**) Experimentally measured responsivities of Ohmic devices. (**d**) Numerically calculated photocurrent response (equation (2)) for the Schottky devices. (**e**) Numerically calculated absorbed power (equation (1)) for the Ohmic devices using an *l*_MFP_=25 nm.

We provide further evidence that the nonresonant photocurrent is from interband transitions by comparing the wavelength-dependent photocurrent responsivity of plasmonic nanowires with either a Schottky or Ohmic interface. Scanning-electron microscope images of the different wire widths are shown in Fig. 3a. No attempt was made to optimize the nanowires for maximum responsivity. The increase in photocurrent for wavelengths shorter than 410 nm is due to direct absorption in TiO_2_, corresponding to a bandgap of 3.03 eV^30^. For Schottky devices, the responsivity shows strong polarization dependence (Fig. 3b). For TE polarization, the photocurrent response shows unambiguous resonances corresponding to plasmonic modes of the nanowires. The broad resonances for the small nanowire widths correspond to dipolar plasmon modes, whereas the sharp resonances for nanowire widths of 155 nm and larger are quadrupolar plasmon modes (Supplementary Fig. 5). TM-polarized excitations produced little to no photocurrent, because the excitation is detuned from any plasmon mode.

Since the Ohmic devices can collect low-energy electrons excited via interband transitions, we expect a rise in the photocurrent for photon energies of the incident light above 2.3 eV, where the interband transitions in gold begin to occur. We verify using photocurrent maps (Supplementary Fig. 6) that the photocurrent at shorter wavelengths is localized to the metal nanostructure and does not correspond to absorption in the TiO_2_. Ohmic devices (Fig. 3c) show the predicted increase and also show that the responsivity matches very well with the absorption spectrum (Fig. 3e) calculated using equation (1), which includes interband transitions. In particular, the response at shorter wavelengths closely follows the onset of interband transitions as it manifests in the imaginary component of the gold dielectric permittivity (Supplementary Fig. 7). Overall, the Ohmic devices show a damped photocurrent response when illuminated on resonance, which results from damping by the Ti-barrier layer. We note that the photocurrent for plasmon excitation (TE) in the Ohmic devices is also strongly enhanced compared with the TM excitation and reaches a similar magnitude as for direct excitation at the interband threshold at much higher energy. This shows that plasmon-induced carrier generation indeed is an efficient process. Although the Ohmic devices have a thin Ti layer between the antenna and the substrate, preventing a direct comparison of the photocurrent responsivity of the two types of devices, the responsivity of Ohmic and Schottky devices at the plasmon resonances are similar in magnitude, suggesting that most of the plasmon-induced hot electrons have sufficient energy to traverse the Schottky barrier.

## Discussion

Through theoretical modelling, we show that plasmon-induced hot-carrier generation is independent of interband carrier generation. Since the inherent material absorption is described by the imaginary part of the dielectric, the calculated absorbed power equation (1) will include interband transitions. In contrast, hot-carrier formation from plasmon decay is predicted to be determined directly by the plasmon-induced local electric field |**E**(**r**)|^*2*^ (ref. 22) To model the contribution to the photocurrent of carriers from plasmon decay, we thus integrate the field-intensity enhancement over the volume *V*_MFP_:


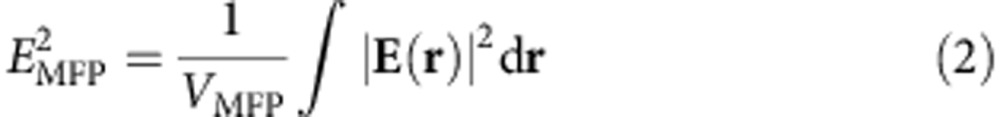


Using this method, we obtain excellent agreement between the calculated (Fig. 3d) and measured (Fig. 3b) photocurrent responsivity for the Schottky devices. We note that the enhancement for our devices is strongest at the metal–semiconductor interface (Supplementary Fig. 8) and that all integrations for these devices were performed within one mean-free path of the interface (25 nm)^34^. Previous work^9^,^10^,^11^,^16^ has shown that increasing the field enhancement near the interface is important for increasing the efficiency of hot electron devices. Since the photocurrent response matches with the field-intensity enhancement rather than with the material-dependent absorption, we have shown that plasmonic hot-carrier generation occurs independently of material absorption.

It is important to note that we do not observe a Fowler-type response for the Schottky devices. In general, Fowler theory is used to explain a quadratic increase in the photocurrent responsivity for higher-photon energies^21^ and derived using a quadratic density of states, equal probability of excitation for all states and an isotropic momentum distribution for excited carriers. Our result indicates that one or more of these assumptions is not likely to be applicable to plasmonic carrier generation. It is unclear why this work and other experiments with rutile TiO_2_ (refs 15, 17) do not observe a Fowler-type response but we speculate that it could be related to the behaviour of indirect semiconductors like Si or anatase TiO_2_ (ref. 35), for which absorption also increases quadratically near the band edge^36^. On the contrary, rutile TiO_2_ has a direct bandgap, which results in an absorption coefficient that increases sharply at the band edge. Therefore, all measured photocurrent is directly attributed to surface plasmons and independent of any absorption in the semiconductor substrate.

**Figure 4:**
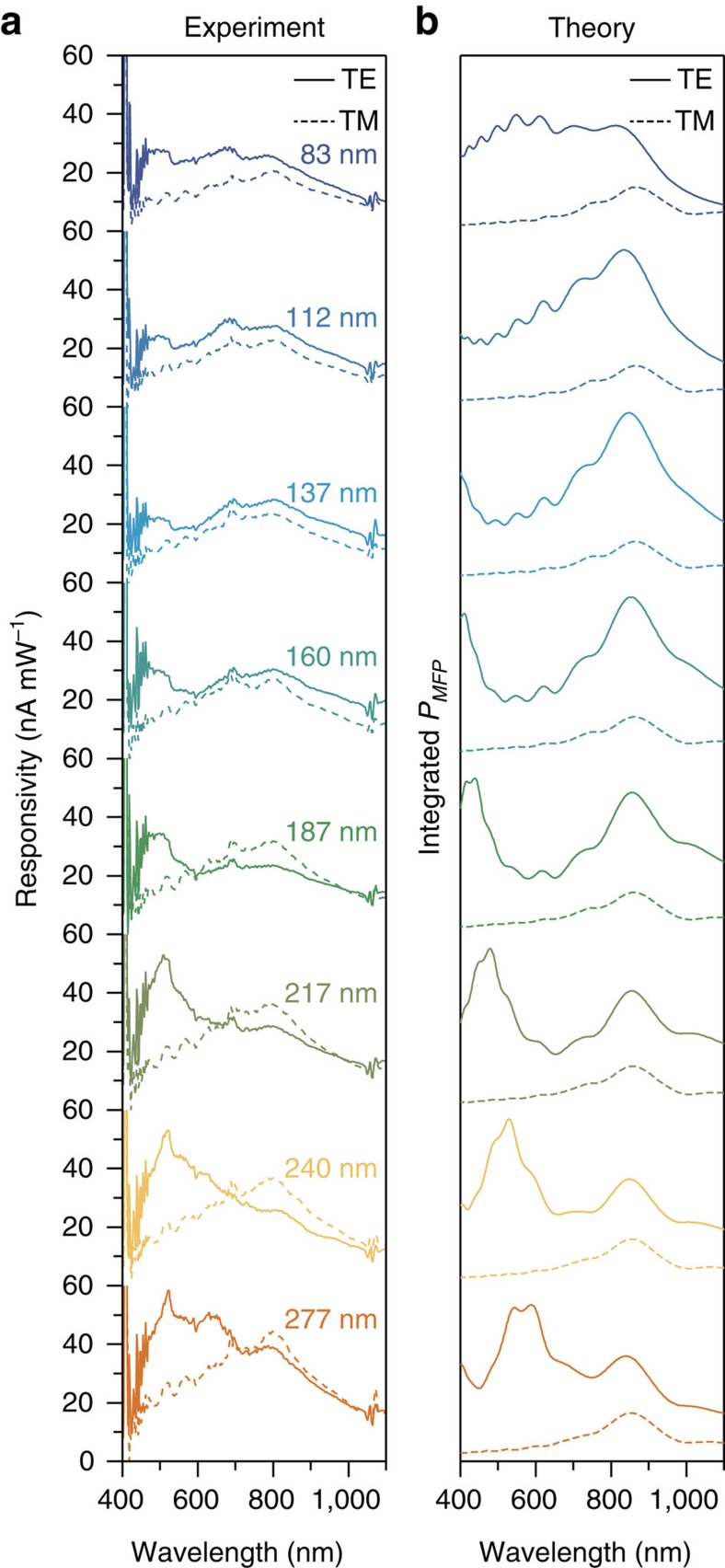
Hot Carriers in aluminium nanowires. (**a**) Photocurrent responsivities of Ohmic aluminum structures. The peak at ∼800 nm in both TE and TM polarizations corresponds to aluminum interband transitions. (**b**) Numerically calculated absorbed power within one mean-free path of the interface (18 nm)^34^ for a single nanowire.

By comparing gold Ohmic devices with equivalently fabricated aluminium Ohmic devices, we establish that the Ohmic devices collect hot carriers generated from interband transitions. One major difference between gold and aluminium is that aluminium interband transitions occur at a much longer wavelength, near ∼800 nm (∼1.5 eV)^37^. Therefore, we predict that Ohmic aluminium devices will exhibit a peak at 800 nm for both TE and TM polarizations. Our measured responsivities, shown in Fig. 4a, confirm this prediction. Theoretical modelling reproduces most features of the experimental results (Fig. 4b). We note that the theoretical calculations likely overestimate the intensity inside the nanostructure, which leads to a peak at ∼850 nm, as opposed to the interband peak at 800 nm (Supplementary Fig. 9). We did not measure significant photocurrent generation in aluminium Schottky devices. We speculate that the relatively thick Au barrier layer (6 nm) which was required to form a continuous film significantly damps the optical response.

In summary, by comparing the photocurrent generation through plasmon excitation and from direct excitation in simple Schottky and Ohmic devices, we have demonstrated that plasmonic hot-carrier generation results in higher-energy electrons than direct excitation. We have shown that for the Schottky device, the photocurrent responsivity can be calculated by integrating the electric field enhancements over a volume within the electronic mean-free path of the surface of the plasmonic nanoparticle. For the Ohmic device, the responsivity can be calculated by integrating the imaginary part of the metallic permittivity over the same volume and is dominated by interband transitions. Our results open up new avenues for increasing photo-conversion efficiency through the collection of both plasmonic and interband photocurrent and could find broad applicability in novel optoelectronic devices.

## Methods

### Sample fabrication

As described in more detail in the Supplementary Methods, TiO_2_ samples (Princeton Scientific) are first cleaned by sonicating in IPA for 5 min. The samples are then transferred to a high-vacuum chamber and baked in an alumina-coated molybdenum boat (Mathis) at ∼1,200 °C for 90 min. Next, plasmonic nanostructures are fabricated using standard e-beam lithography techniques. Structures were written in 8 × 10 arrays in poly-methyl-methacrylate (PMMA A4 495K, MicroChem) and developed for 60 s in a 1:3 solution of methyl-iso-butyl-ketone:isopropanol solution. Different device types (for example, Au Schottky, Au Ohmic, Al Ohmic) were fabricated on the same substrate by sequential evaporation and shadow masking. Au films were deposited at 0.5 Å s^−1^; Al films were deposited at 0.7 Å s^−1^; and Ti films were deposited at 1 Å s^−1^. The base pressure for all evaporations was 5.0 e−7 Torr or better. Liftoff was performed at 60 °C in PG Remover solution (MicroChem). Finally, a large Ti contact pad was deposited and the sample was mounted onto a glass microscope slide using cyanoacrylate.

### Optoelectronic measurements

We use a lock-in amplifier (Signal Recovery 7280) for responsivity measurements. We performed current-voltage measurements using a Keithley 2400 Picoammeter. The samples were illuminated using a broadband white light laser source (Fianium). Specific wavelength bands were selected using an acousto-optic tunable filter (Crystal Tech). Photocurrent responsivity spectra were obtained using two acousto-optic tunable filter crystals, one tuned for the visible region (400–700 nm) and another crystal tuned for the near-infrared region (700–1,100 nm). The light is focused onto the sample using a 20 × long-working distance objective (Mitutoyo) with a numerical aperture of 0.42. Individual devices were contacted using nickel-plated tungsten probes (Picoprobe). We found greater variability in the photocurrent measurements for aluminium devices (Supplementary Fig. 10).

### Theoretical modelling

We simulated the optical response of the nanostructures using a commercial finite difference time domain software package (Lumerical). For simplicity, we studied the response of a single nanowire of infinite length. This approximation is justified by the fact that we find no significant differences in the calculated spectra when simulating a single nanowire or an array of nanowires (Supplementary Fig. 11). In all calculations, the sizes of the nanowires were chosen to be identical to the fabricated structures. Furthermore, the corners of the nanowires were slightly rounded to avoid numerical instabilities and match the experimental conditions more precisely. The aluminium devices were simulated with a 3 nm oxide layer. The dielectric functions of the different materials were taken from tabulated data: Au from ref. 38, Al and Al2O3 from ref. 39 and TiO2 from ref. 40. The incident light was modelled as a plane wave with the polarization transverse (TE) or parallel (TM) to the orientation of the nanowires and propagation normal to the substrate. Perfect-matched layers were used as boundary conditions to simulate the infinite substrate and absorb scattered light. All calculations have been converged to ensure the reliability and accuracy of the simulation results.

## Additional information

**How to cite this article:** Zheng, B. Y. *et al.* Distinguishing between plasmon-induced and photoexcited carriers in a device geometry. *Nat. Commun.* 6:7797 doi: 10.1038/ncomms8797 (2015).

## Supplementary Material

Supplementary Information Supplementary Figures 1-11 and Supplementary Methods
