# Supplementary material for: Distinguishing between plasmon-induced and photoexcited carriers in a device geometry
Source: Nat Commun. 2015 Jul 13;6:7797. doi: 10.1038/ncomms8797 (PMC4510964; doi:10.1038/ncomms8797)
Supplement: Supplementary Information — Supplementary Figures 1-11 and Supplementary Methods [file ncomms8797-s1.pdf]

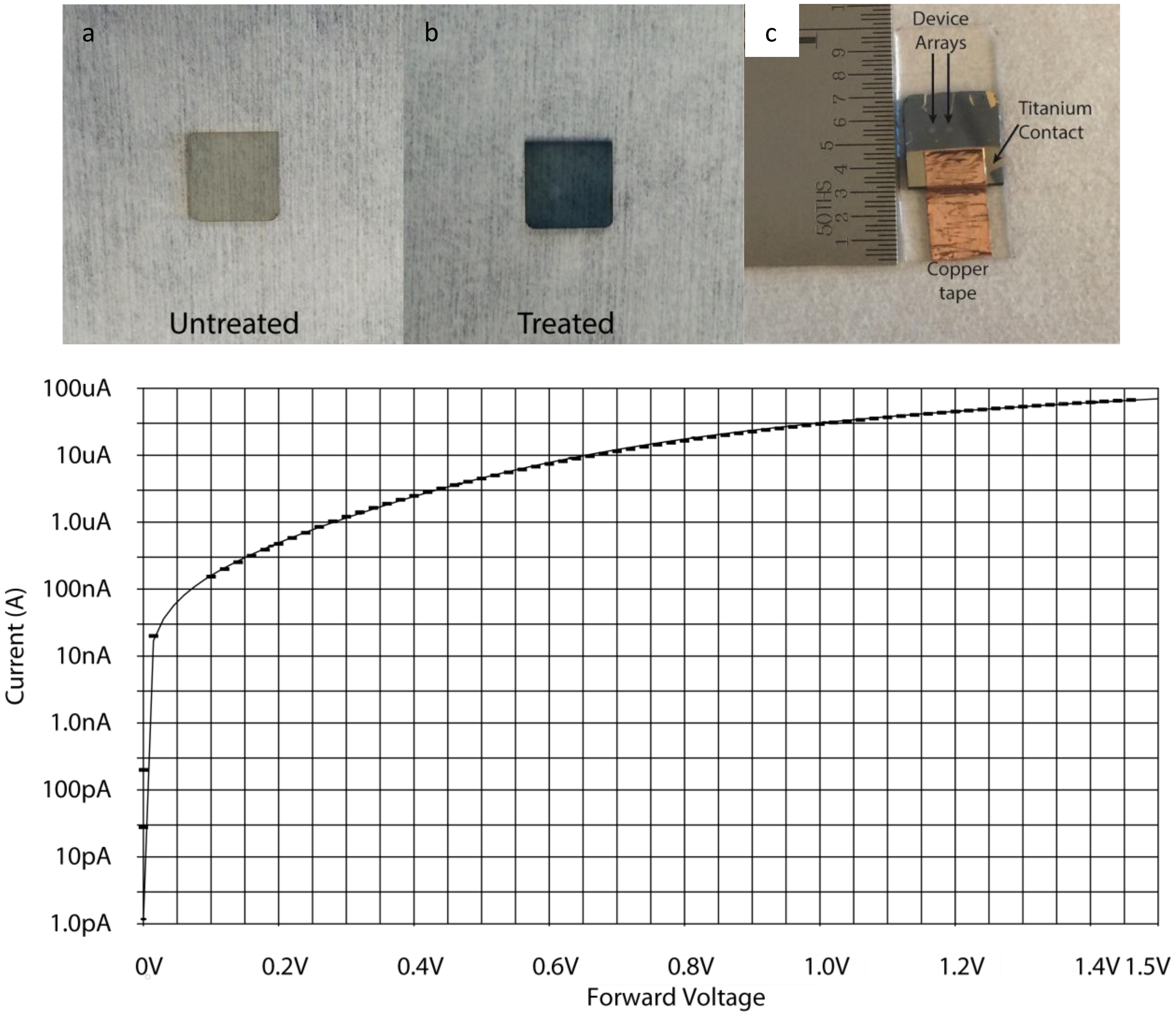

Supplementary Figure 1

Sample images and Schottky IV Fits

(a) Picture of a rutile (10 mm x 10 mm x 1 mm) <100> TiO2 substrate as purchased and (**b)** after heat treatment **(c)** final device after all fabrication steps. The device arrays show aluminum ohmic devices on the left and gold ohmic devices on the right. The device is mounted on a glass slide. The adjacent ruler is 1 inch for scale. **(d)** Fit (line) to IV data (dash) for forward bias using PSpice circuit simulator. Fit parameters: A* = 1100 A/cm^2/K, A = 40^2 μm + 20*25*0.175 nm, T = 300 K. Calculated barrier height: 1.03 eV, series resistance: 10.1 KΩ, Ideality factor: 4.9.

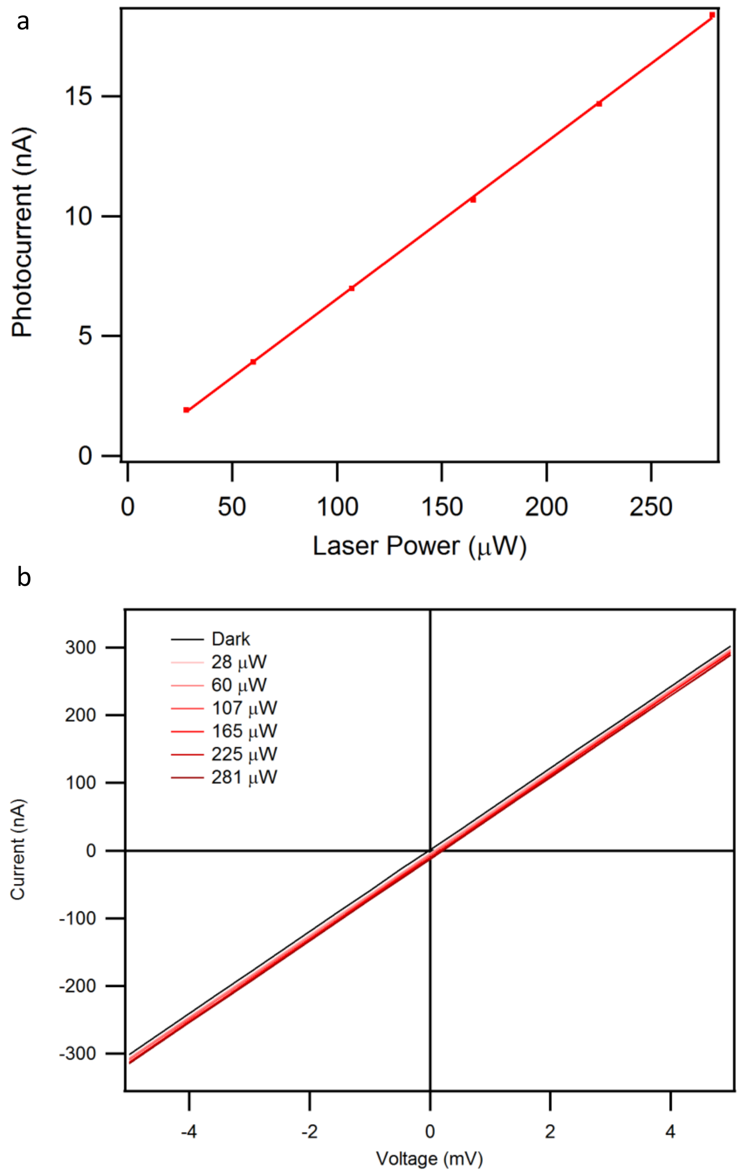

Supplementary Figure 2

IV curves of different illumination powers

(a) Power-dependence of photocurrent in an ohmic device. The structure is illuminated on-resonance with 675 nm laser light. The photocurrent is measured using a lock-in amplifier (Signal Recovery). The linear dependence shows that the photocurrent is not due to any nonlinear effect. Line is best fit to measured data. **(b)** Small signal current-voltage (IV) curves under different illumination powers. The laser is focused at the center of a nanowire array, where each finger is 82 nm. The excitation wavelength is 600 nm and TE polarized. The IV curves show a constant downward shift with increasing laser intensity, consistent with charge injection into the semiconductor. If the observed photocurrent was the result of changes in device conductance or junction resistances, the slope of the IV curves would increase with increasing laser power.

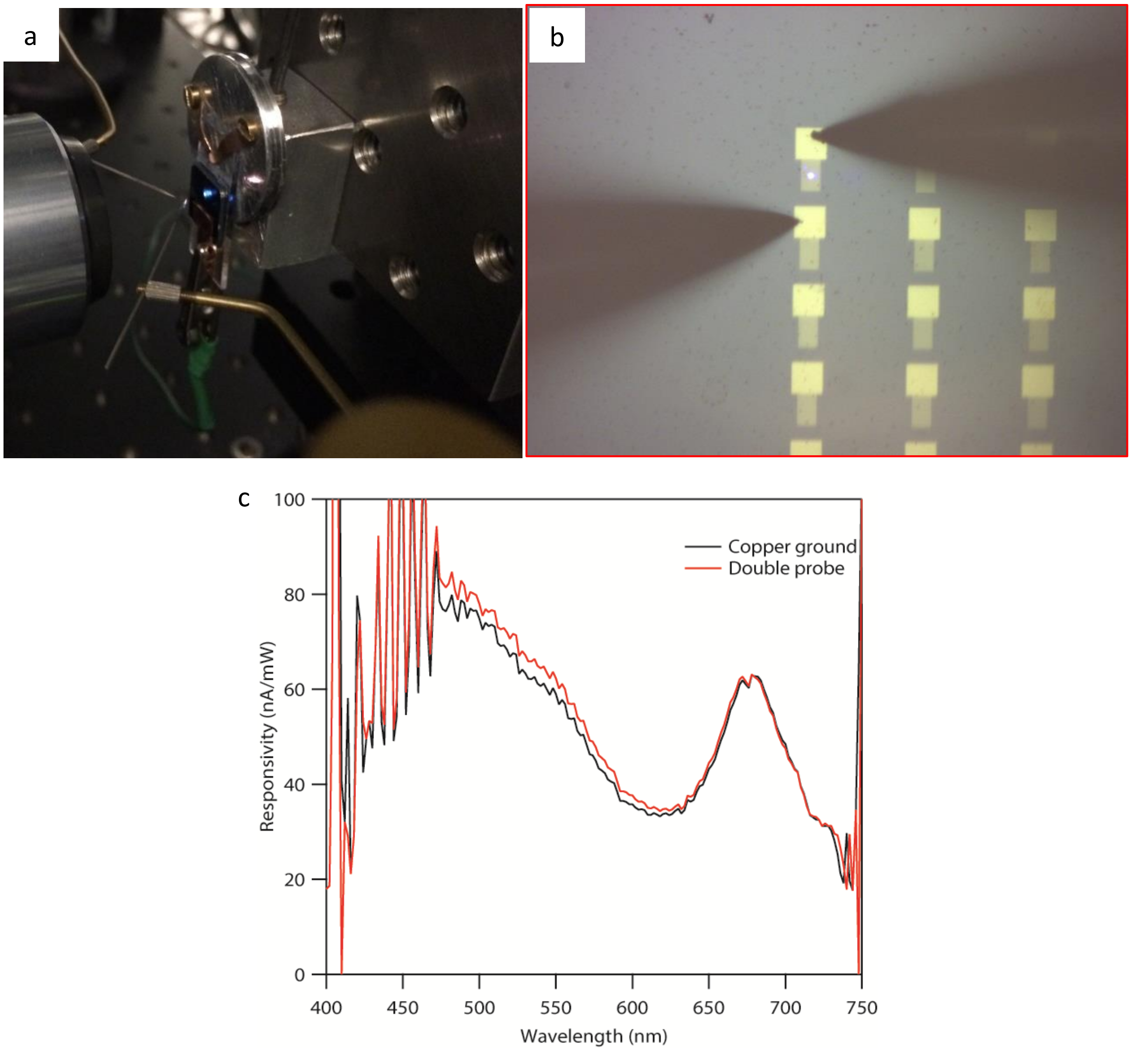

Supplementary Figure 3

Losses due to carrier recombination

(a) Photograph of a sample mounted in the photocurrent setup in the standard measurement configuration. All measurements in the paper were performed using this mounting setup and the copper tape as a ground electrode. The distance from the copper tape to the devices is approximately 1.5 mm. **(b)** Measured device responsivity using two microprobes. The devices are separated by a 5 μm gap. **(c)** The two measured photocurrents using either the copper ground (black) or using a probe ground (red).

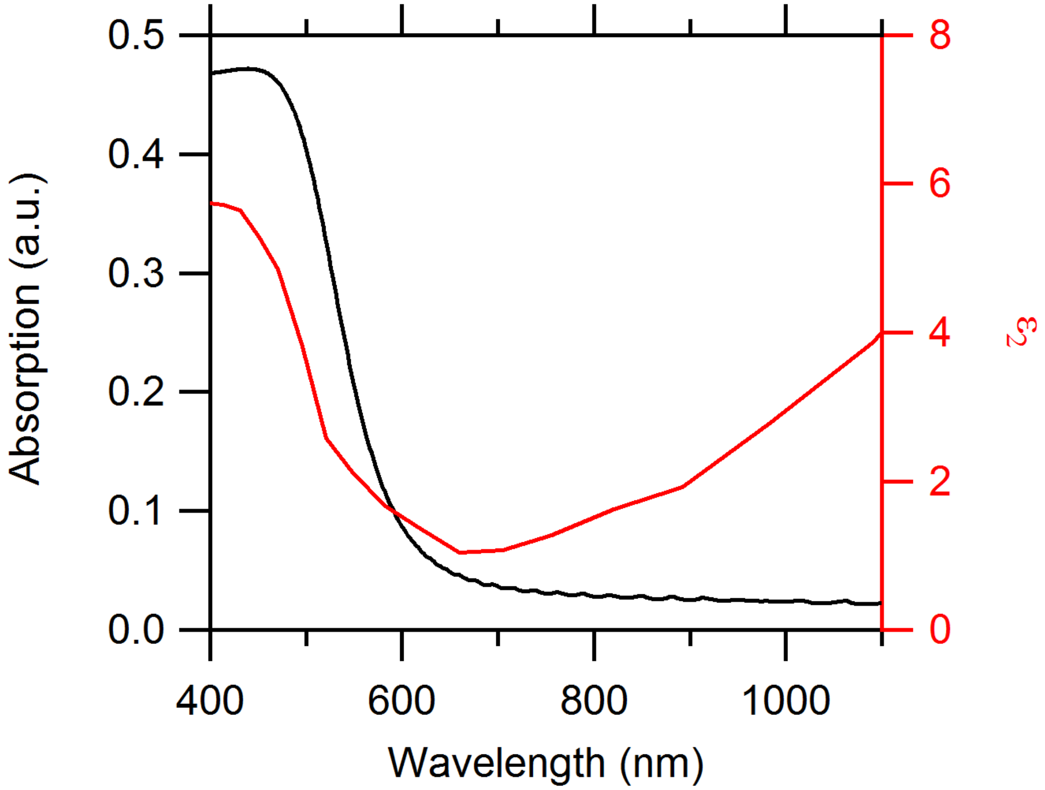

Supplementary Figure 4

Calculated pad absorption

Calculated absorption of the pad. The pad was simulated using a 2D geometry. A plane wave excitation propagates normal to the substrate. The simulated metal pad is 50 nm tall by 20 μm wide. The ripples in the absorption spectra is likely due to numerical instability. The increase in absorption for short wavelengths (< 600 nm) is due to interband transitions and not a plasmonic resonance.

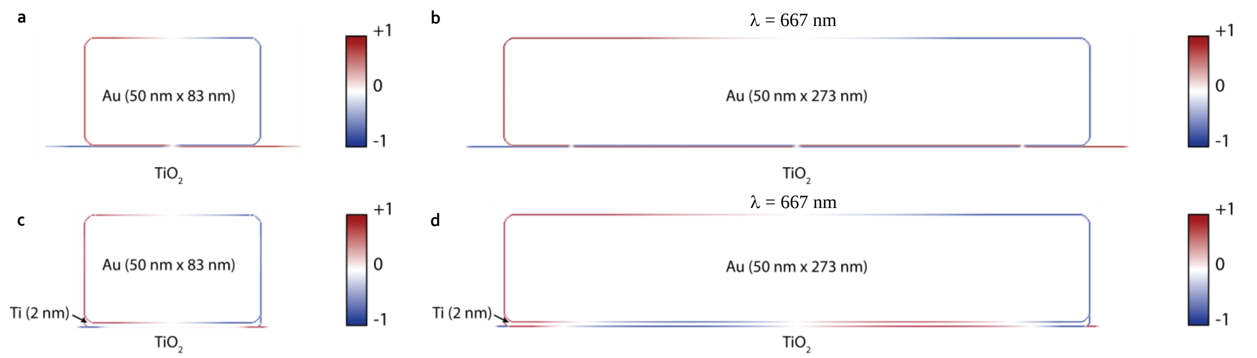

Supplementary Figure 5

Schottky and Ohmic Device Charge Plots

Charge plots for an Au/TiO_2 type device with a nanowire width of **(a)** 83 nm and **(b)** 273 nm. The 83 nm wire clearly exhibits a dipolar plasmon resonance while the larger 273 nm wire exhibits a quadrupolar resonance. Charge plots for an ohmic (Au/Ti/TiO_2) type device with nanowire widths of **(e)** 83 nm and **(f)** 273 nm. The titanium barrier layer is 2 nm. All simulations were performed at the resonant wavelength.

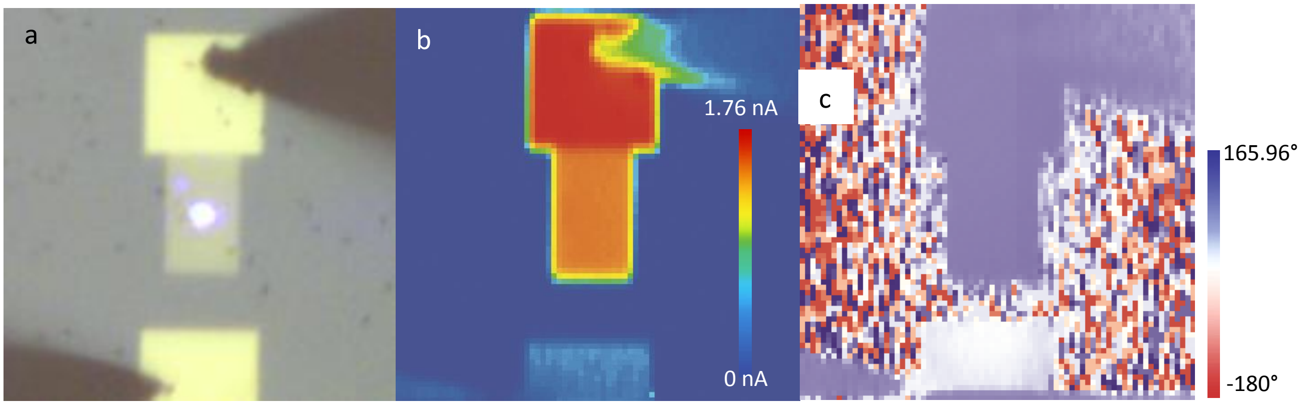

Supplementary Figure 6

Short wavelength photocurrent maps

(a) Bright-field image of a fabricated device, illuminated with 450 nm light. The light is TE polarized. **(b)** The corresponding photocurrent map and **(c)** the measured phased map. The bottom probe is disconnected electrically but physically touching the sample.

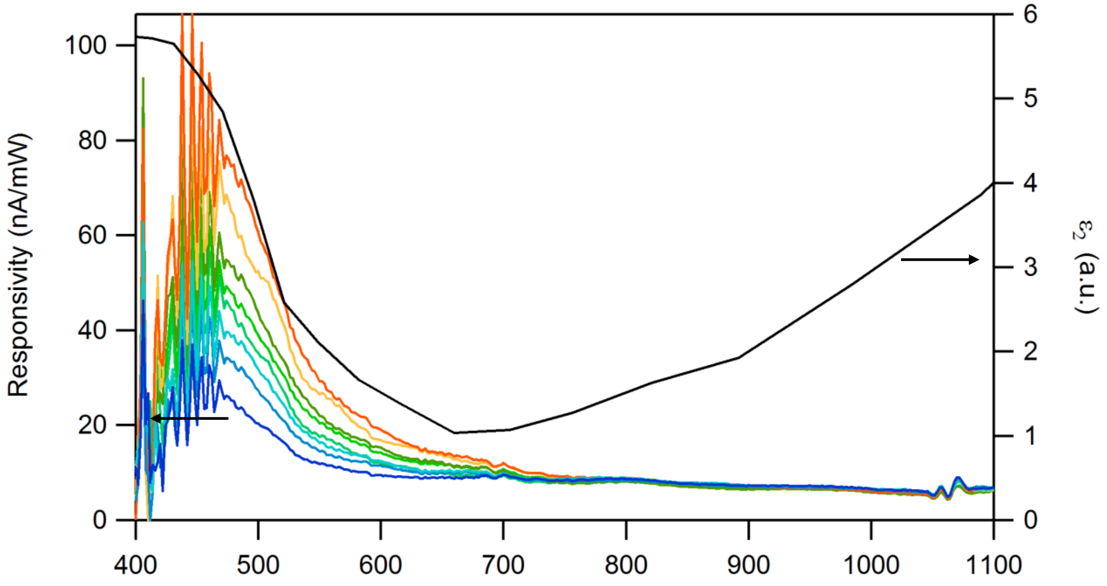

Supplementary Figure 7

Gold Dielectric Function vs Responsivity

Device responsivities plotted versus imaginary part of the gold dielectric function (Johnson and Christy). The device responsivities are the same as those reported in Figure 2d for TM polarization. The imaginary part of the gold dielectric increases for wavelengths shorter than 650 nm due to interband absorption. The nanowire responsivities only correlate well with this spectral portion of the dielectric function, indicating that the photocurrent generation in the nanowires originates from interband transitions in gold. While it is possible that some electrons are excited from deeper in the electronic band structure up to the Fermi level, this carrier generation mechanism is negligible relative to carrier generation from interband transitions.

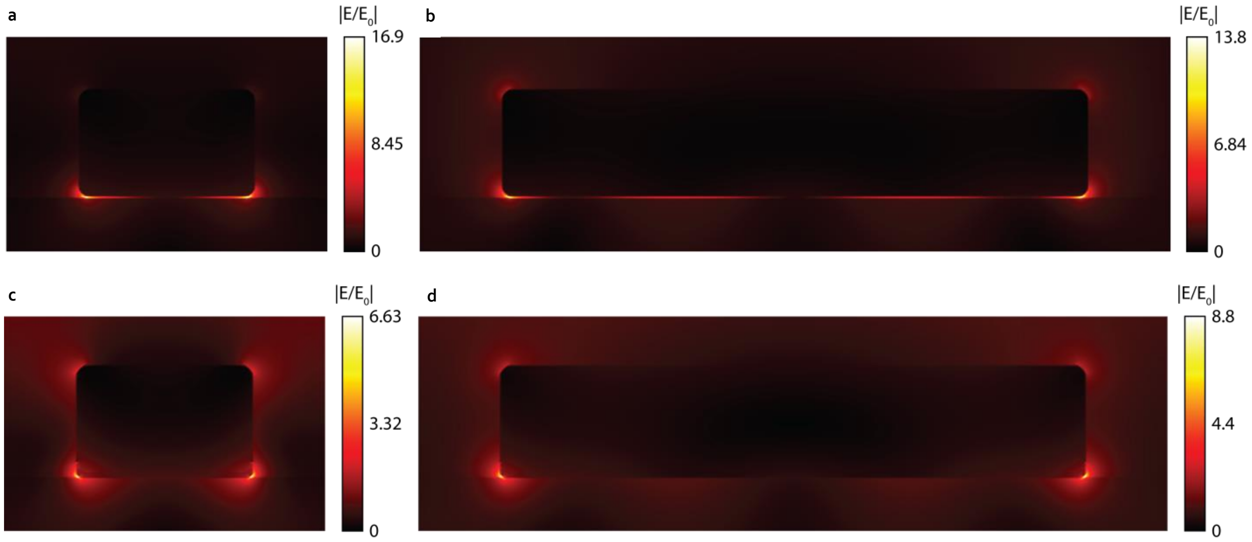

Supplementary Figure 8

Field and Charge Plots

Field plots for the Schottky devices with nanowire widths **(a)** 83 nm and **(b)** 273 nm. Field plots for ohmic devices with wire widths of **(c)** 83 nm and **(d)** 273 nm. The ohmic devices have a 2 nm Ti barrier layer. All field plots show the maximum field localization at the metal-semiconductor interface.

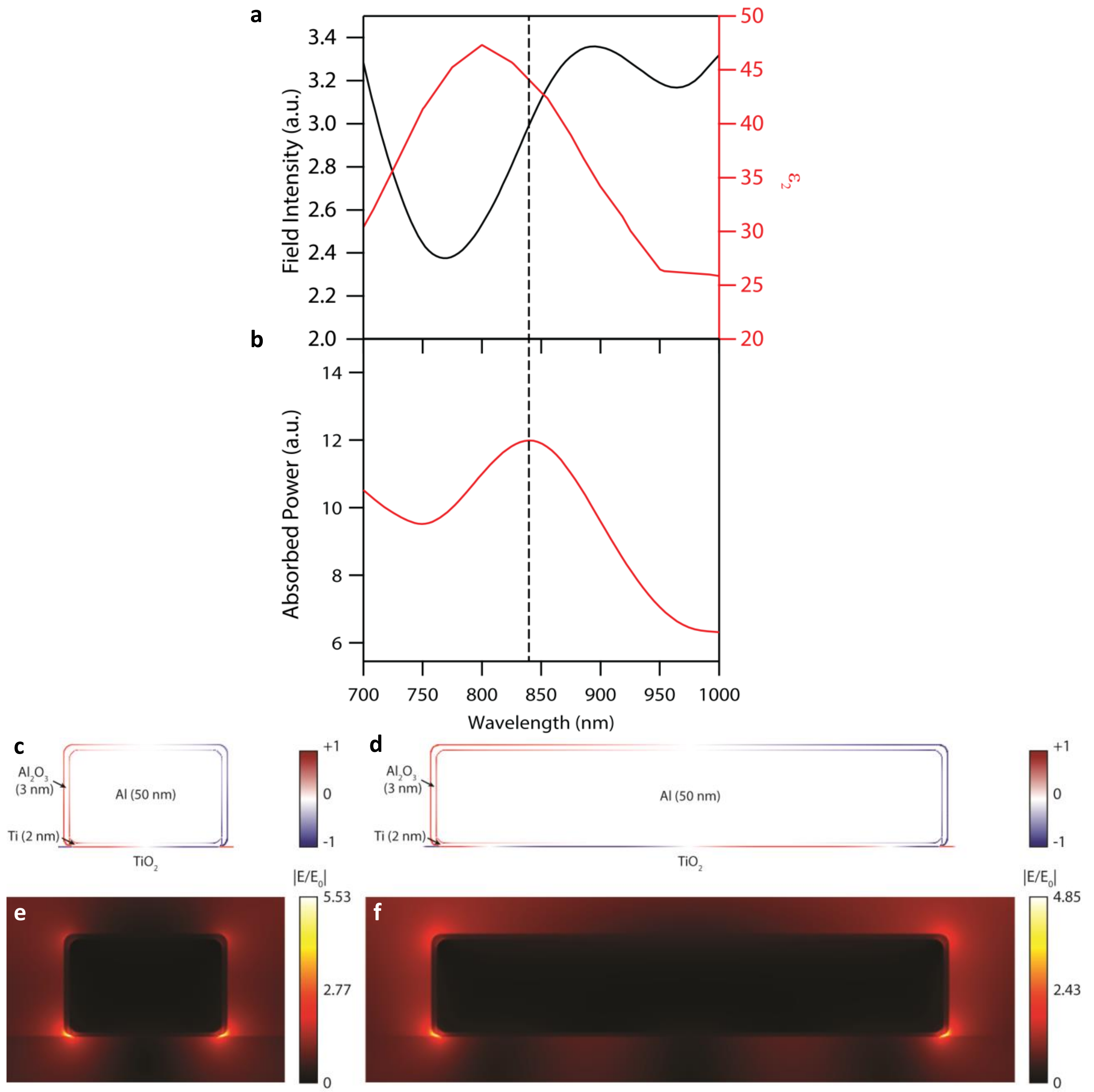

Supplementary Figure 9

Calculated Absorbed Power and Field Intensity for An Al Nanostructure

(a) Plot comparing the imaginary part of the dielectric function (red) and field intensity (black). **(b)** The calculated absorbed power. The peak at 850 nm in the calculated absorbed power is due to contributions from both the imaginary part of the dielectric as well as the field intensity. Since this peak is not observed, simulations likely overestimate the field intensity inside the metal. Charge (**c**, **d**) and field plots (**e, f**) of the 82 nm and 273 nm aluminum ohmic structures.

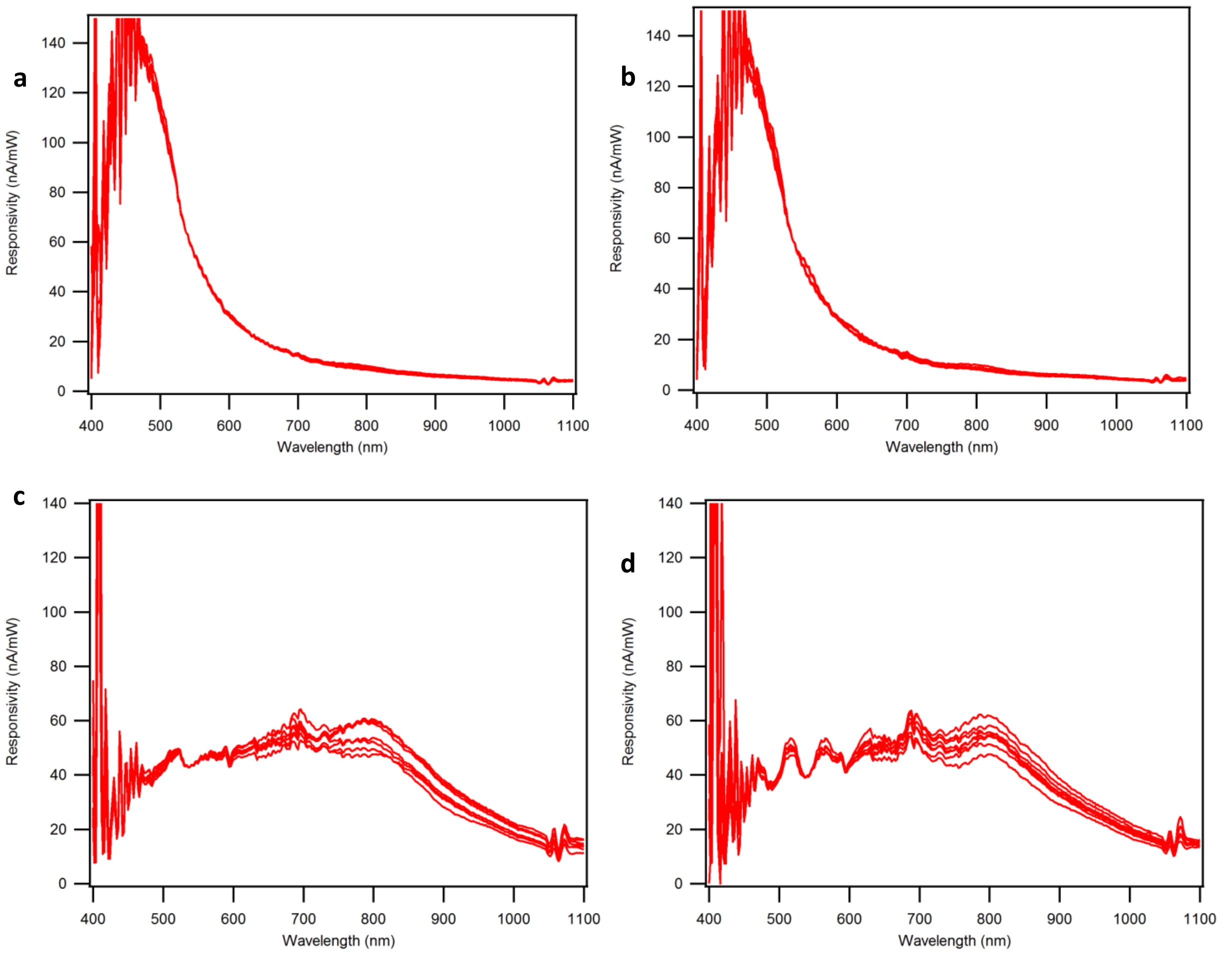

Supplementary Figure 10

Pad responsivities

Pad responsivities of all gold devices under **(a)** TE and **(b)** TM illumination. Pad responsivities of aluminum ohmic devices under **(c)** TE and **(d)** TM illumination. All device responsivities are shown. The increased variability in the aluminum devices is likely due to the small oxide layer that forms between the microprobe and the device pad.

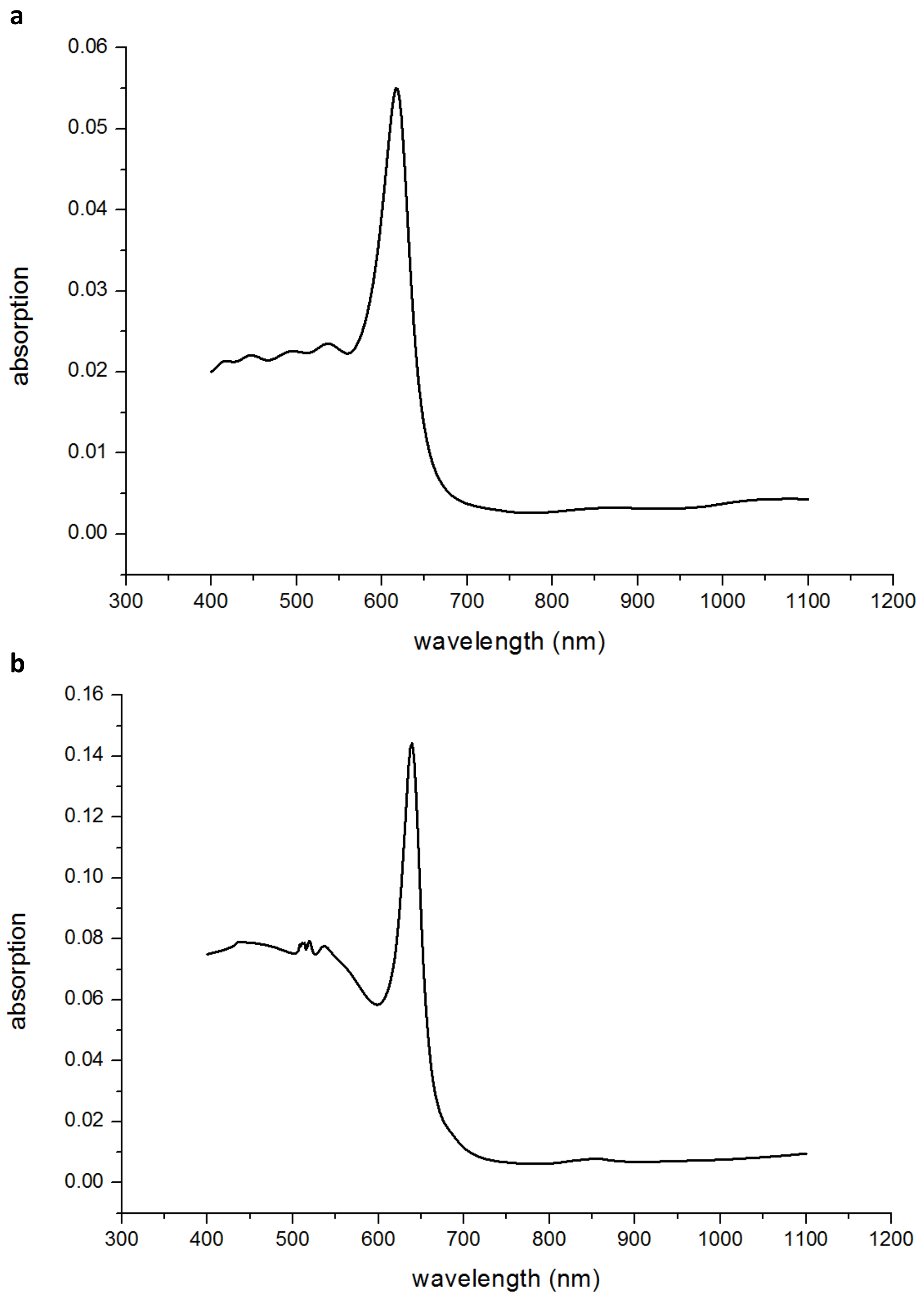

Supplementary Figure 11

Calculated absorption in single wire vs wire array

Calculated absorption in a **(a)** single wire vs **(b)** a wire array for a nanowire width of 209 nm. The spectra were calculated using normal incidence plane-wave illumination and identical simulation geometries. The only difference is that the wire array simulations use a periodic boundary condition of 500 nm, corresponding to the experimental device pitch.

Supplementary Methods – Sample Fabrication

1. Sonicate TiO_2 substrate in isopropyl alcohol (IPA) for 5 minutes
2. Place in vacuum chamber (Base pressure 1e-6 T) and heat (~1200 °C) in an alumina-coated molybdenum boat (Mathis) for 90 minutes. The temperature was increased from room temperature to the final baking temperature over the course of 5 minutes. The measured resistance across the heat treated sample is ~10 kΩ.
3. Blow dry with N_2 gas
4. Spin coat Microchem 495K PMMA A4 at 3000 RPM for 60 s
5. Write patterns using electron beam lithography (beam current: 41 pA, spot size = 2.5 nm). Separate each pattern by at least 500 μm (for shadow masking). For this paper, each nanostructure is written ten times in a single vertical column. There are 8 different nanostructure wire widths resulting in an 8 X 10 array. Each column is separated by 50 μm.
6. Develop in MicroChem 3:1 IPA:MIBK developer solution for 60 s
7. Use shadow mask to cover 2 of 3 arrays
8. Evaporate 50 nm Au at 0.5 Å/s
9. Move shadow mask to cover 2 other arrays
10. Evaporate 2 nm Ti at 1 Å/s
11. Evaporate 50 nm Al at 0.7 Å/s
12. Repeat shadow masking/evaporation for ohmic aluminum structures (2 nm/50 nm Ti/Al)
13. Lift-off in PG Remover solution (heated to 60 °C) for 60 min
14. Blow dry using N_2
15. Use shadow mask to cover arrays
16. Evaporate 100 nm Ti (1 Å/s) for large ohmic contact to the substrate
17. Bond sample to glass slide using cyanoacrylate and dry for 10 min
18. Apply copper tape (See finished device)
